# Fishers’ Perceptions of Fishing Dynamics and Socio-environmental Threats in Coastal Protected Areas of Northeastern Brazil

**DOI:** 10.1007/s00267-026-02465-6

**Published:** 2026-04-16

**Authors:** Yedda Christina Bezerra Barbosa de Oliveira, Priscila Fabiana Macedo Lopes, Tiago Almeida de Oliveira, Diogo Guedes Vidal, Fátima Alves, Maria do Rosário Tomás Rosa, José da Silva Mourão

**Affiliations:** 1 Programa de Pós-Graduação em Etnobiologia e Conservação da Natureza, Universidade Federal Rural de Pernambuco, Dois Irmãos, Recife, Pernambuco Brazil; 2 Centre for Functional Ecology (CFE), Universidade de Coimbra Calçada Martim de Freitas, Coimbra, Portugal; 3 Departamento de Ecologia, Universidade Federal do Rio Grande do Norte Campus Universitário, Lagoa Nova Natal, Natal, RN Brazil; 4 Research Institute of the University of Bucharest University of Bucharest Bucharest, Bucharest, Romania; 5 Institute of Biological Research Cluj National Institute of Research and Development for Biological Sciences Cluj-Napoca, Cluj-Napoca, Romania; 6 Departamento de Estatística, Universidade Estadual da Paraíba, Bairro Universitário, Campina Grande, Paraíba Brazil; 7 Department of Social Sciences and Management, Universidade Aberta Rua da Escola Politécnica, Lisboa, Portugal; 8 Departamento de Biologia, Universidade Estadual da Paraíba, Bairro Universitário, Campina Grande, Paraíba Brazil

**Keywords:** Conservation threats, Livelihoods, Marine governance, Coastal communities, Small-scale fisheries

## Abstract

Small-scale fisheries are central to the economy, food security, and cultural continuity of many coastal communities across the Global South, yet fishing activities and community well-being are increasingly exposed to pressures from overfishing, pollution, and coastal ecosystem degradation. When fishing occurs within or near coastal protected areas, regulatory frameworks and livelihood dependence become tightly intertwined, making fishers’ perceptions of the environment and fishing dynamics a socially structured dimension of these systems rather than merely individual views. We interviewed 105 fishers from three coastal protected areas in Northeastern Brazil (Paraiba and Pernambuco) to (1) analyze their perception of changes in small-scale fisheries and socio-environmental threats, and (2) examine how socioeconomic factors (e.g. sex, education, income, dependence on fishing) influence these perceptions. We did a content analysis of the qualitative interview data and applied multinomial logistic regression to model their perception of socio-environmental threats. Our findings showed that male fishers were significantly more likely to perceive pollution (odds ratio [OR] = 5.45) and overfishing (OR = 2.57) as major threats. Additionally, higher income was associated with a lower likelihood of perceiving overfishing (OR = 0.27) and pollution (OR = 0.009) as significant concerns, regardless of gender. Lower income levels were associated with greater sensitivity to socio-environmental threats, while gendered divisions of labor shaped distinct environmental perceptions. These findings demonstrate that socio-ecological dynamics in coastal protected areas are structured by poverty and social inequalities. Effective governance must therefore integrate biodiversity conservation with strategies that address livelihood security, gender inequities, and structural vulnerability in small-scale fisheries.

## Introduction

Small-scale fisheries represent a vital component of the economy and livelihoods of numerous coastal communities, particularly in the Global South, where natural resource use is closely tied to subsistence practices and household income (da Silveira and Ferreira 2024; Chambon et al. 2024). However, coastal ecosystems are increasingly burdened by drivers such as overfishing, pollution, and climate change—pressures that are widely recognized by coastal communities (Viegas et al. 2016; Martins et al. 2018; Mudge 2018; Sandoval Gallardo et al. 2021). These drivers not only contribute to the decline of fish stocks but also threaten the long-term sustainability of small-scale fisheries and the well-being of the communities (Sowman and Sunde 2018).

Perceptions are understood as subjective interpretations through which individuals observe, make sense of, and evaluate environmental conditions, actions, or outcomes (i.e., socially constructed judgments rather than objective assessments; Bennett 2016). While perceptions may be informed by experience and knowledge, they should not be conflated with scientific or experiential knowledge itself, as they reflect interpretative evaluations shaped by lived realities. From this perspective, variation in perceptions does not necessarily indicate differences in ecological knowledge, but rather differences in how environmental change and management are felt interpreted (Bennett 2016). Although environmental changes and threats to coastal ecosystems have been consistently perceived by small-scale fishers across generations (Martins et al. 2018; Medeiros et al. 2024), the perception of resource conditions and the effectiveness of fisheries management strategies may vary due to individual and social factors (Gehrig et al. 2018; Mudge 2018; dos Santos et al. 2024). Broader social dimensions, including local context, cultural practices, and belief systems, significantly shape fishers’ environmental perceptions and attitudes (Alves et al. 2021). From a political economy perspective, these interpretative processes are deeply shaped by patterns of livelihood dependence, economic vulnerability, access to resources, and institutional marginalization (Béné and Friend 2011). In small-scale fisheries, where livelihoods are closely tied to common-pool resources, such structures condition whether environmental change is perceived as a manageable fluctuation, a threat to subsistence, or a constraint imposed by external development and governance dynamics (Béné and Friend 2011).

Several studies suggest that fishers who are financially dependent on fishing are less likely to comply with regulatory measures (Karper and Lopes 2014) and conservation initiatives targeting threatened species (Vieira et al. 2024). Conversely, younger fishers, who often diversify their livelihoods through alternative activities such as tourism, appear to show greater willingness to adopt management regulations (Karper and Lopes 2014; Silva and Lopes 2015; da Silveira and Ferreira 2024). At the same time, perceptions of declining fish abundance are also more frequently reported among older fishers with long-time fishing experience (Martins et al. 2018; Medeiros et al. 2024). Socioeconomic variables also affect the perception of the benefits and drawbacks of conservation initiatives in communities living in or near protected areas (PAs), as well as awareness of the objectives of these areas (Htun et al. 2012; Chechina et al. 2018). Individuals who report negative attitudes or perceive losses associated with PA management often frame these concerns in terms of economic impacts (Htun et al. 2012).

Cognitive and social psychology offer valuable theoretical frameworks for understanding the challenges individuals face in perceiving gradual environmental changes over time (Pauly 1995; Papworth et al. 2009; Baquiano 2016; Soga and Gaston 2018). One such framework is the shifting baseline syndrome (SBS) (Pauly 1995), which helps explain environmental generational and individual amnesia (Kahn Jr 2002; Papworth et al. 2009; Soga and Gaston 2018). Environmental generational amnesia occurs when each generation grows accustomed to the appearance and conditions of its own environment. In systems undergoing progressive degradation, younger generations fail to recognize the extent of environmental decline experienced by previous generations, leading to distinct baseline perceptions across age cohorts (Kahn Jr 2002; Fernández-Llamazares et al. 2015). In contrast, individual amnesia refers to the tendency of individuals to revise their perception of environmental conditions throughout their lives, often forgetting or downplaying the ecological states they experienced in the past (Essl et al. 2015; Fernández-Llamazares et al. 2015). However, critical perspectives caution that framing shifting baseline syndrome solely as a cognitive or generational phenomenon risks obscuring the role of socio-economic structures and power relations in shaping whose knowledge is recognized and whose environmental histories are marginalized. By privileging scientific reconstructions of past conditions over local ecological knowledge, baseline narratives may unintentionally reinforce epistemic inequalities and overlook the interdependent relationships between traditional communities and ecosystems (Campbell et al. 2009).

Beyond individual memory and experience, environmental perceptions are also socially constructed (Gehrig et al. 2018). They are influenced by interpersonal interactions, cultural beliefs, traditional practices, and historical contexts, all of which contribute to the social representations (e.g., collective understandings shared within a group) about the natural environment (Moscovici 1976).

In coastal Brazil, small-scale fisheries are legally defined as activities primarily dependent on family-based labor and fishing as the main source of livelihood (Brasil 2009). These fisheries operate within complex socio-environmental contexts marked by regulatory overlap in protected areas, limited institutional coordination, persistent poverty, and unequal access to decision-making arenas (Barros 2021). Recognizing the diversity of perceptions in fishing communities is essential for promoting participatory and inclusive governance, as different groups have specific needs and priorities. Fishers’ perceptions of environmental change influence their willingness to engage in conservation practices and comply with regulatory measures (Silva et al. 2021; Vieira et al. 2024). If communities do not perceive threats as severe, there may be resistance to conservation policies, which can undermine their effectiveness.

Despite a growing body of literature on fishers’ perceptions of environmental change, much of this research remains analytically fragmented, often privileging either descriptive qualitative accounts or isolated quantitative predictors, with limited attention to how socioeconomic variables interact to produce distinct perception profiles rather than uniform responses (dos Santos et al. 2024). At the same time, quantitative studies commonly rely on binary or linear outcome structures, which tend to obscure the coexistence of multiple, qualitatively different perception states, such as recognizing specific threats, perceiving generalized environmental decline, or reporting no perceived threat at all. These limitations are particularly consequential in PAs, where regulatory regimes, livelihood diversification, and historical experiences with conservation can generate socially differentiated and non-linear patterns of threat perception, yet management interventions are frequently designed under assumptions of homogeneous problem recognition (Seixas and Kalikoski 2009). Consequently, understanding not only whether fishers perceive environmental change, but how different social groups represent socio-environmental threats remains an underexplored dimension in the literature.

In this context, this study addresses the question: How do fishers perceive the dynamics of fishing over time and the socio-environmental threats they face? By adopting a mixed-methods approach that integrates qualitative analysis with multinomial regression modeling, our objectives were: (1) to analyze the perception of changes in small-scale fisheries by fishers, and (2) to identify how socioeconomic factors may shape the perception of socio-environmental threats.

## Materials and Methods

### Study Areas

A global systematic review of research in marine protected area has shown that conservation initiatives are generally focused on biodiversity and the ecology of non-human species (Borges et al. 2020). In the Brazilian context, however, conservation studies tend to adopt more participatory and socially-oriented approaches, diverging from the dominant global trend (Borges et al. 2020). This orientation can, in part, be attributed to the National System of Protected Areas (Sistema Nacional de Unidades de Conservação—SNUC, Federal Law No. 9.985/2000), which defines two overarching objectives across the twelve categories of PAs: the full protection of biodiversity, and the sustainable use of natural resources, especially in territories traditionally inhabited and used by local communities.

Within this framework, fisheries management in Brazil has evolved through a wide range of participatory institutional arrangements, varying in the degree of involvement of fishers in decision-making processes (Seixas and Kalikoski 2009). In the context of fisheries resource use, governance effectiveness depends on clearly delineating territories of interest, customary practices, and patterns of resource use, particularly by identifying different degrees of dependence on fisheries resources. Such differentiation is central to defining who has access to resources and under what conditions, as well as to addressing conflicts related to access and property rights (Seixas et al. 2011). Evidence from different coastal and inland contexts shows that, even where legislation formally prescribes limited participation, management practices often incorporate consultative, shared, or advisory forms of co-management. However, these arrangements remain uneven and frequently fragile, with deliberative councils and shared governance instruments still in an embryonic stage in many PAs, reflecting persistent asymmetries of power between the State and traditional fishing communities (Seixas and Kalikoski 2009).

This study focuses on three Protected Areas (PAs) categorized under SNUC as sustainable use protected areas: Environmental Protection Areas (Áreas de Proteção Ambiental – APAs) and Extractive Reserves (Reservas Extrativistas – RESEXs) (Table 1). Although both types aim to promote sustainable resource use, they differ in emphasis. APAs are primarily designed to conserve biodiversity in the context of land-use planning and regional development, whereas RESEXs seek to safeguard the cultural heritage and livelihoods of traditional populations (BRASIL 2000). The PAs addressed in this study include two APAs, Barra do Rio Mamanguape (APABRM) and Tambaba State Environmental Protection Area (APAT), and one RESEX, the Acaú-Goiana Extractive Reserve (REAG). The three coastal PAs are located in the states of Paraíba and Pernambuco, in northeastern Brazil.

**Table 1:** Governance arrangements of the protected areas, distribution of interview participants, and their fishing activities

Coastal protected area	Jurisdiction	Council structure	Established (year)	Number of surveys (%) (*N* = 105)	Main fisheries operated
Acaú-Goiana Extractive Reserve (REAG)	National	Deliberative	2002	47 (45)	Shellfish gathering; lobster fishing
Barra do Rio Mamanguape Environmental Protection Area (APABRM)	National	Advisory	1993	32 (30)	Gillnet fishing; shellfish and crab gathering
Tambaba Environmental Protection Area (APAT)	Regional	Advisory	2002	26 (25)	Gillnet fishing; lobster fishing

Established in 1993, the APABRM (6.776°S, 34.926°W) aims to protect critical ecosystems and species, including the West Indian manatee (peixe boi marinho - *Trichechus manatus manatus*), mangrove forests, remnants of the Atlantic Forest, and freshwater resources (Perazzo et al. 2013; Medeiros et al. 2023). The area encompasses the Mamanguape River Estuary, spanning approximately 16,400 hectares and comprising 32 small villages. Most residents along the riverbanks are small-scale fishers, whose livelihoods have traditionally depended on fishing (Mourão and Nordi 2018).

Created in 2002, APAT (7.417°S, 34.917°W) covers about 3,270 hectares across the municipalities of Conde, Pitimbu, and Alhandra. It was established to conserve coastal ecosystems, support territorial planning, and preserve scenic landscapes associated with tourism. Since 2005, parts of the coastal zones in Conde and Pitimbu have been legally designated for sea turtle conservation, overlapping with traditional small-scale fishing territories (Almeida 2006).

The REAG (7.566°S, 34.837°W), created in 2007, emerged from demands by traditional extractive communities in the municipalities of Pitimbu and Caaporã (Paraíba state) and Goiana (Pernambuco state) (Fadigas and Garcia 2010). Covering 6,676.62 hectares, the reserve includes six communities (Baldo do Rio, Tejucupapo, Povoação de São Lourenço, Carne de Vaca, Acaú, and Caaporã) (Rodrigues et al. 2017), whose residents engage in small-scale fishing and the harvesting of estuarine invertebrate resources such as the Venus clam (marisco - *Anomalocardia flexuosa*), and the crabs (caranguejos - *Callinectes danae*, *Goniopsis cruentata*, *Ucides cordatus*, and *Cardisoma guanhumi*). Historically, conflicts have arisen between these communities and industrial shrimp farming operations that have occupied areas within the Goiana River estuary particularly on Ilha do Tariri, previously used by traditional extractive populations (Fadigas and Garcia 2010).

None of the three PAs included in this study has an approved management plan (REAG) or systematically updated official records on the number of active small-scale fishers (APABRM and APAT). The absence of consolidated institutional data reflects long-standing challenges in monitoring and governance across these PAs.

### Data Collection

We conducted a mixed-methods study involving 105 small-scale fishers from the three studied coastal PAs. Semi-structured, face-to-face interviews were carried out with adult fishers (aged 18 and older) of these PAs, who voluntarily agreed to participate in the study (see Ethics statement below). Participants were identified either during extractive activities (e.g., at fishing or shellfish harvesting sites) or through snowball sampling (Goodman 1961). While snowball sampling may introduce selection bias by over-representing more socially connected individuals, its use in this study was limited and complemented by direct field-based recruitment to reduce this effect.

During fieldwork, interviews were conducted with small-scale fishers identified as female or male based on observable biological characteristics and contextual social markers, rather than self-identification. All participants responded to a unified interview protocol, irrespective of sex. Although gender diversity beyond the binary was not a central focus, the inclusion of both male and female fishers aimed to reflect distinct lived experiences within predominantly male fishing communities (Heidari et al. 2016; Freitas et al. 2020).

Data collection took place over a period of 20 days in each PA: in February, April, and July 2023 at APABRM, REAG, and APAT, respectively. Participant observation was employed as a complementary method to enrich data collection and strengthen trust between the lead researcher and participants. By engaging in everyday activities and informal interactions, the researcher reduced social distance and created conditions for more open, spontaneous, and contextualized exchanges (Albuquerque et al. 2014).

Open-ended questions were used during the interviews to reduce social desirability bias, defined as the tendency to overstate intentions in alignment with perceived social norms or expected pro-environmental behaviors (Juvan and Dolnicar 2016; Dean and Wilson 2023). Interviews were conducted in the presence of a community facilitator, who assisted with communication and, when authorized by the interviewees, took photographs of the activities (see Figs. S1, S2, and S3). Community facilitators did not influence interview responses; their role was limited to facilitating access, supporting communication, and helping establish trust between the researcher and participants. The interview protocol was structured into two main sections: (1) socioeconomic characteristics (e.g. sex, age, education level, fishing experience, sources of income, monthly household income), and (2) perceptions of environmental and fishing dynamics (e.g. perceived environmental and fishing-related changes over time, shifts in fishing effort and gear use, and restrictions on species access and use). Monthly income data were expressed in Purchasing Power Parity (PPP) dollars and calculated by converting the Brazilian real to PPP dollars as of December 17, 2024. To refine the interview protocol, three pilot interviews were conducted; however, data from these interviews were not included in the final analysis.

### Ethics Statement

This research was approved by the Ethics Committee of the Federal Rural University of Pernambuco (#5.897.834) and by SISBio (Biodiversity Authorization and Information System) (#81328-4), granting permission to conduct fieldwork within PAs and with traditional communities. Participation in the study was entirely voluntary. All participants were informed about the objectives of the research, the procedures involved, and the intended use of the data prior to participation. Informed consent was obtained from all participants, who were assured of the anonymity and confidentiality of their responses. Participants were also informed that they could refuse to answer any question or withdraw from the study at any time, without any consequences, including after the interviews had been completed. This research was informed by the Sex and Gender Equity in Research (SAGER) guidelines, which advocate for the systematic consideration of sex and gender in studies involving human subjects (Heidari et al. 2016; Van Epps et al. 2022).

### Data Analysis

#### Categorizing Perception of Environmental and Fishing Dynamics and Threats

The analysis followed a fully inductive, two-phase qualitative approach. In the first phase, all responses from the semi-structured interviews were fully transcribed and subjected to manifest content analysis (Bardin 2011), focusing on the explicit statements made by fishers regarding perceived changes in small-scale fishing. Through repeated readings, responses were grouped into main themes reflecting broad dimensions of perceived change (e.g. changes in fish abundance, fishing effort, technology, or access to resources).

In the second phase, a latent content analysis was conducted to interpret the underlying meanings associated with these perceived changes, allowing the identification of a second analytical layer corresponding to perceived socio-environmental threats (Bengtsson 2016). These threats were not predefined in the interview script but emerged inductively from participants’ narratives, often extending beyond strictly environmental aspects to include social, economic, and conservation-related concerns. Examples of main themes that emerged include overfishing, pollution, and food insecurity.

**Fig. 1:**
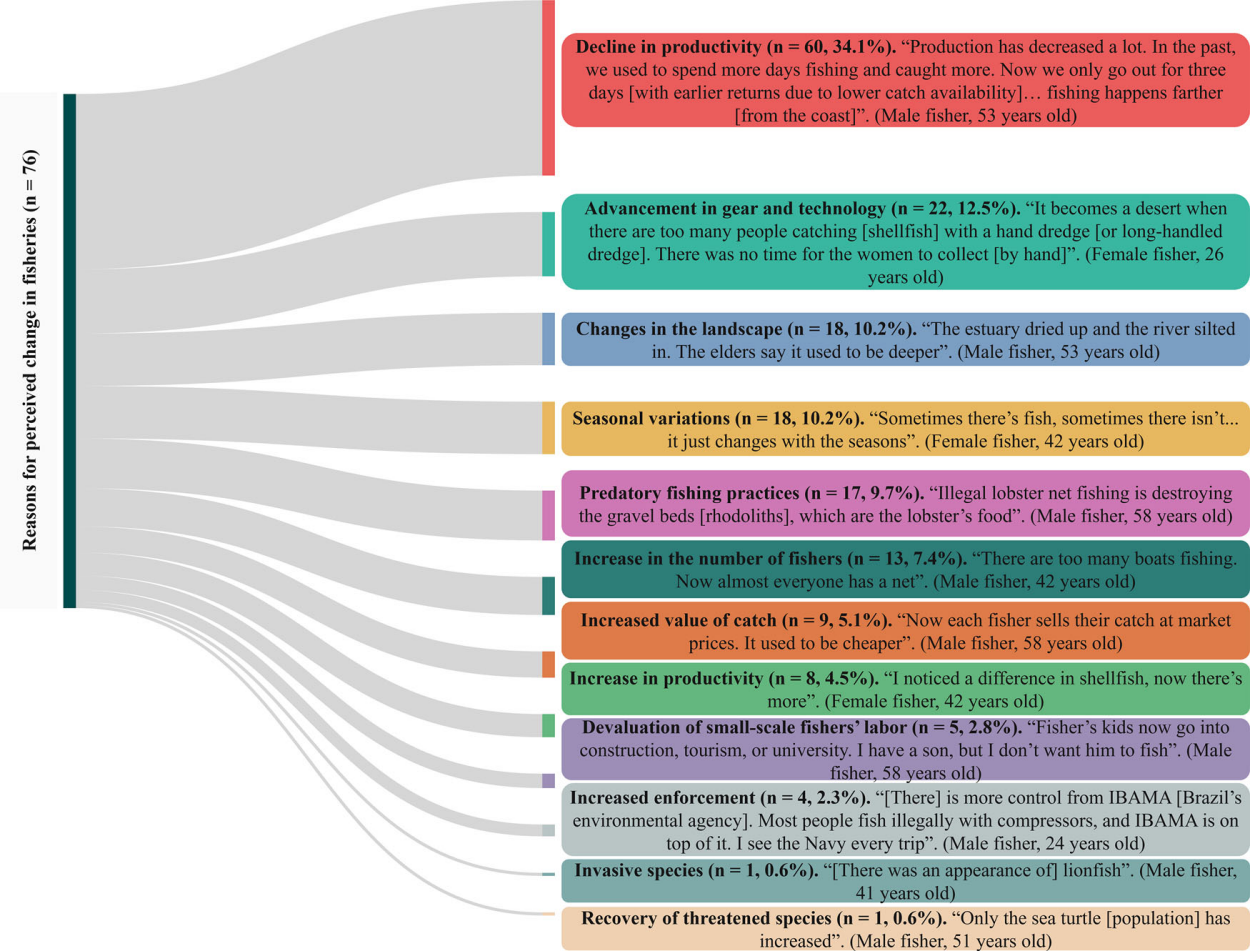
Sankey diagram illustrating the perceived changes in environment and small-scale fishery. Flows represent the relative proportion (%) of response units derived from fishers’ open-ended statements and grouped into main themes. As individual respondents could report more than one perceived change, multiple response units may originate from a single interview (*n* = 176). Quotes are used to illustrate fishers’ perception for each theme

**Fig. 2:**
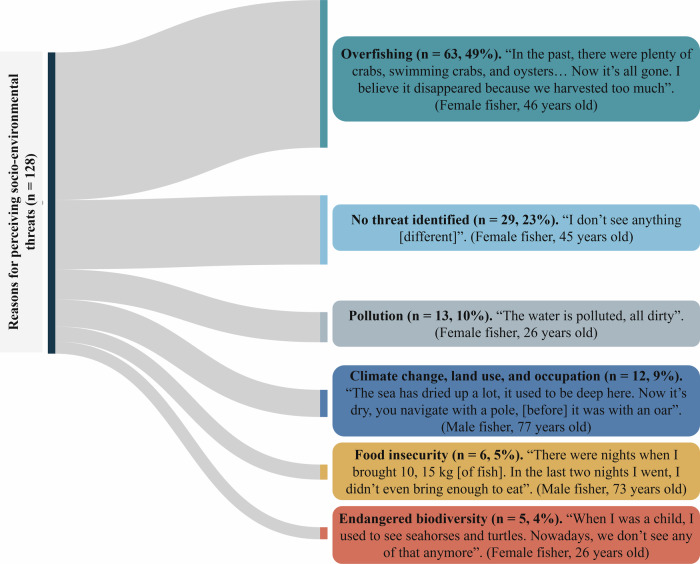
Sankey diagram illustrating the perceived socio-environmental threats affecting small-scale fishery (n = 128). Flows represent the relative proportion (%) of response units derived from fishers’ open-ended statements and grouped into main themes. As individual respondents could report more than one perceived change, multiple response units may originate from a single interview *n* = 128*). Quotes are used to illustrate fishers’ perception for each theme

All main themes identified in first phase (perceived changes in small-scale fishing) and second phase (perceived socio-environmental threats) are presented in Figs. 1 and 2, respectively.

Coding was performed independently by two researchers and subsequently cross-validated through comparison to assess convergence; discrepancies were resolved through discussion until consensus was reached. The unit of analysis corresponded to individual response units rather than to respondents. Although 105 interviews were conducted, participants could mention more than one issue within a single answer, resulting in multiple response units derived from the same interview. Each response unit was assigned to a single main theme within each analytical phase.

The two analytical phases were conducted independently, generating distinct qualitative datasets. The coded material was compiled into a spreadsheet, and relative frequencies were calculated based on the total number of response units within each phase.

### Statistical Analysis

A multinomial logistic regression model was employed to analyze the socioeconomic factors associated with fishers’ perceptions of different socio-environmental threats, which were treated as the response variable. Five categories of perceived threats were defined: (1) overfishing, (2) pollution, (3) climate change, land use and occupation, (4) endangered biodiversity, and (5) food insecurity. An additional category, (6) no identified threat, was created to serve as the reference category in the model.

The explanatory variables included: age, years of fishing experience, household size, and fishing frequency per month (all treated as continuous variables); sex and dependence on fishing as the primary source of income (binary variables); and education level and monthly household income (categorical variables). The PA was not included as an explanatory variable, as comparing governance regimes across areas was beyond the analytical scope of this study and the sample size within each PA did not support robust between-group comparisons.

Multinomial logistic regression, estimated using the maximum likelihood method, is appropriate for modeling outcomes with more than two unordered response categories. It allows for the estimation of the probability of each outcome relative to a reference category (Hosmer et al. 2013; Agresti 2018). The model uses the *logit* link function, which transforms response probabilities into log-odds, facilitating interpretation of the resulting coefficients (McCullagh & Nelder, 1989). The general form of the multinomial logistic model is given by:

$$\begin{array}{c}{\rm{P}}({\rm{Y}}={\rm{k}})=\tfrac{\exp ({\mathrm{X\beta }}_{{\rm{k}}})}{{\sum }_{{\rm{j}}=0}^{{\rm{K}}}\exp ({\mathrm{X\beta }}_{{\rm{j}}})},\,\,\,\,\,{\rm{k}}=1,2\ldots ,{\rm{K}}\end{array}$$

where ${\boldsymbol{X}}$ represents the values of the explanatory variables, and ${\boldsymbol{\beta }}$ corresponds to the estimated coefficients of the model.

- ${\boldsymbol{P}}\left({\boldsymbol{Y}}={\boldsymbol{k}}\right)$: probability that the dependent variable ${\boldsymbol{Y}}$ falls into category ${\boldsymbol{k}}$.
- ${\bf{\exp }}({\boldsymbol{X}}{{\boldsymbol{\beta }}}_{{\boldsymbol{k}}})$ exponential of the product between the vector of explanatory variables ${\boldsymbol{X}}$ and the coefficient vector ${{\boldsymbol{\beta }}}_{{\boldsymbol{k}}}$ for category ${\boldsymbol{k}}$.
- ${\sum }_{j={0}}^{K}exp(X{\beta }_{j})$: sum of the exponentials of all ${\boldsymbol{X}}{{\boldsymbol{\beta }}}_{{\boldsymbol{j}}}$ products across the ${\boldsymbol{K}}$ categories, ensuring the probabilities are properly normalized.

The analysis was performed using the *nnet* package in R software, version 4.2.3 (R Core Team 2023). The estimated coefficients for each threat category represent changes in the log-odds of perceiving a specific threat relative to perceiving no threat (Long & Freese, 2014).

To validate the model and determine the best fit, we applied the Akaike Information Criterion (AIC), which allows for comparison among models with differing numbers of parameters (Greene, 2018). The selection of the reduced model was based on the exclusion of non-significant predictors, as indicated by *p*-values (*p* > 0.05) and odds ratios (OR > 1: increases the likelihood of the event occurring; OR = 1: no association; OR < 1: decreases the likelihood of the event). Only predictors with statistically significant effects were retained in the final model.

To capture potential associations between monthly income and the perception of socio-environmental threats, we included linear, quadratic, and cubic terms specifically for this variable. The linear term accounts for proportional changes in threat perception as income increases. The quadratic term allows for curvilinear relationships, capturing non-constant changes in effect. The cubic term accounts for more complex variations, potentially identifying shifts in the direction of the relationship between income and threat perception.

We also applied an equivalence index to quantify the number of similar or overlapping responses among fishers, which ranged from one to four perceived threat categories (out of a total of six). This allowed us to adjust the proportions of perceived threats across all interviewed fishers.

## Results

Participants were predominantly male (64.8%, *n* = 68), while female accounted for 35.2% of the sample (*n* = 37). In terms of age, the majority ranged from 40 to 66 years old (80%; *n* = 84; SD = 12.59). A significant portion of the fishers reported extensive fishing experience, with 87% (*n* = 91) having worked in the activity for over 27 years. Regarding educational attainment, 71.4% (*n* = 75) were either illiterate or had completed only primary education.

Fishing was the primary source of household income for 80% (*n* = 84) of respondents, often supplemented by governmental aid (71%; *n* = 75), including retirement benefits, social assistance programs, and other welfare support. Additional income sources included public service or temporary jobs (37%; *n* = 39), and informal work in the tourism sector (12%; *n* = 13). Nearly half of the participants (47.6%; *n* = 50) reported a monthly household income ranging from PPP USD 250.00 to 500.00. On average, households were composed of three members (μ = 3.4 ± 0.72), and respondents reported engaging in fishing activities an average of 17 days per month (μ = 17.35 ± 5.86).

### Perceptions of changes in fishing dynamics and threats

When asked about perceived changes in fishing activity over time, respondents provided 12 distinct types of responses (Fig. 1), primarily highlighting threats related to resource availability and broader transformations in fishing dynamics. Their responses suggest an ongoing process of environmental degradation, often associated with interlinked environmental, technological, and social changes.

Although some narratives reflect a degree of resilience in adapting to these changes, the predominant perception is that small-scale fishing has undergone a process of socio-ecological reconfiguration, which directly compromises its long-term viability.

Based on the interpretation of fishers’ open-ended responses, six categories of perceived socio-environmental threats were identified (Fig. 2). These reflect a shared perception that fishing practices themselves have contributed to the depletion of fish stocks, creating a cycle of increasing vulnerability. Although not all respondents recognized immediate risks, several narratives expressed growing concern about the impacts of human pressures on coastal, marine and estuarine ecosystems and local livelihoods.

### Effect of Socio-economic Factors on Perceived Threats

Male respondents showed a higher perception of threats in comparison to female respondents, while monthly family income had a negative and significant effect, with the perception of threats decreasing as income increased (Table 2).

**Table 2:** Multinomial logistic regression results showing the association between socio-economic factors and the perception of socio-environmental threats

Socio-environmental threats	Independent variables							
	Sex		Linear Monthly Income		Quadratic Monthly Income		Cubic Monthly Income	
	Odds ratio	*p* value	Odds ratio	*p* value	Odds ratio	*p* value	Odds ratio	*p* value
Overfishing	2.567	0.063	1.007	0.992	0.277	**0.021**	0.610	0.305
Pollution	5.458	**0.032**	0.000	0.000	0.000	0.000	0.009	0.000
Climate change, land use and occupation	3.582	0.209	0.000	0.000	0.000	0.000	0.007	0.000
Biodiversity	5.855	0.144	0.000	0.000	0.000	0.000	0.007	0.000
Food insecurity	NA	0.874	0.008	0.988	0.000	0.968	0.065	0.980

Only significant variables are shown

Statistically significant values are highlighted in bold

In the overfishing category, males were more likely to perceive this threat than female (odds ratio [OR] = 2.57, *p* = 0.063). The analysis of income indicated that higher income levels are associated with a significant decrease in the perception of overfishing (OR = 0.27, *p* < 0.05).

For pollution, males also showed a positive association with the perception of this threat (OR = 5.45, *p* < 0.03). Income had a negative effect, with significant *p*-values for the quadratic and cubic terms (*p* < 0.05). These results indicate that the relationship between income and the perception of pollution is not linear. As income increases, the likelihood of perceiving pollution as a threat tends to decrease, but the strength of this effect varies across income levels. Rather than reflecting a constant decline, the influence of income differs in intensity depending on the income bracket, indicating that individuals at different income levels interpret socio-environmental risks in distinct ways.

Except for pollution and a marginal effect for overfishing, the effect of sex was not statistically significant across the remaining threat categories, indicating no detectable gender differences in those perceptions. This lack of statistical significance indicates that, for most threat categories, female and male fishers reported similar perceptions within the limits of the model.

With regard to endangered biodiversity, gender showed no significant effect (OR = 5.85, *p* = 0.14). However, income showed a significant negative association (*p* < 0.05), indicating that higher income levels reduce the perception of this threat, as well as for climate change, land use, and occupation.

For climate change, land use and occupation, the data do not suggest that the perception of these threats differs between male and female fishers (*p* > 0.05). As there were few mentions of the food insecurity threat (*n* = 6), the model did not provide satisfactory results for this category.

Finally, the variables age, years of fishing experience, fishing frequency per month, education level, household size, and dependence on fishing as the primary source of income had no significant effect on perceived threats (*p* > 0.05).

## Discussion

Our study analyzes the perceptions of fishers regarding changes in small-scale fishery practices and socio-environmental threats, contributing to the understanding of how factors such as income and gender influence the formation of different interpretations of the environment. We interpret these perceptions in relation to small-scale fishers’ livelihoods, socioeconomic vulnerability, and governance contexts within protected areas. Understanding how the perceptions of small-scale fishers are formed and structured is essential for identifying the challenges faced by resource users and for strengthening compliance with regulatory measures (Bennett 2016). Furthermore, knowledge about fishers’ perceptions can inform the development of management and conservation strategies for fishery resources, taking into account territorial contexts and the specificities of local communities (Mcalvay et al. 2021; Bennett et al. 2021). It is important to highlight that traditional conservation models, often focused on species and area-based approaches, may be socially unjust and ineffective, which further reinforces the need to adapt public policies and conservation actions to local realities (Burbano and Meredith 2020; McClanahan and Abunge 2020).

### Environmental Change, Technological Shifts, and Perceived Threats

Fishers’ perceptions of changes in small-scale fishery practices reflect the paradox of reconciling economic viability and fishing efficiency with environmental sustainability. On the one hand, there has been increasing access to and development of fishing technologies aimed at enhancing fishing efficiency (Marchal et al. 2007; Torres-Irineo et al. 2014). On the other hand, a long-term decline in fishing productivity has also been observed and reported by fishers here and elsewhere (Teh and Sumaila 2007; Martins et al. 2018). These technological advances have not only optimized the capture of target species but also reduced the fishing effort required. An example includes the adoption of tools such as the long-handled dredge (named *gadanho de cabo*) and the use of plastic crates for shellfish size sorting (locally known as *galea*) (Fig. S2E and S2F), which have replaced manual collection and improved the efficiency of shellfish gathering, reducing the time required for these activities. However, it is important to highlight that there has also been an increase in the number of fishers using these tools, which can result in greater pressure on fishing stocks and lead to long-term unsustainable or predatory practices, as reported in the interviews.

Our findings reflect a critical global trend: the continued decline of fishing stocks in various parts of the world. This scenario is exacerbated by the fact that approximately 80% of global catches originate from fisheries that are unmonitored or have insufficient data (Costello et al. 2012), which significantly compromises sustainable management and evidence-based policy-making. In this regard, it is crucial that technological advances in the fishing sector, even if minor and manual, as detected here, be accompanied by adaptive and participatory management strategies that incorporate local knowledge and directly involve fishers in stock assessments and decision-making processes to ensure both ecological and economic viability of small-scale fishery (Castello et al. 2009; Tallman et al. 2019). Solely relying on techno-scientific solutions is insufficient in the face of the complexity of socio-ecological systems; it is necessary to integrate science, participatory governance, and social justice (Castello et al. 2009; Bennett et al. 2021).

Overfishing was identified as the main reason for the perception of socio-environmental threats among respondents from the studied coastal PAs. Our findings are in line with the existing literature, which shows that small-scale fishers often perceive coastal ecosystem degradation (Aldon et al. 2011), generational declines in catch volume (Panagopoulou et al. 2017; Martins et al. 2018; Medeiros et al. 2024), and attribute these changes to factors such as industrial and illegal fishing, increased fishing effort, and weak regulatory enforcement in the fisheries sector (Aldon et al. 2011; Martins et al. 2018).

Conversely, the absence of perceived threats was the second most common response among fishers. Several factors may explain this outcome. One possible reason is that many fishers view changes in species abundance as naturally seasonal, indicating an understanding of the reproductive and migratory cycles of the species with which they are familiar. This knowledge also reflects their ability to predict variations in resource availability throughout the year for both subsistence and commercial species (Medeiros et al. 2018), reinforcing the argument that local ecological knowledge should be incorporated into fisheries management strategies (Tallman et al. 2019; Pita et al. 2019). However, the fact that seasonality is an aspect recognized by fishers may hinder their ability to distinguish between expected seasonal fluctuations and long-term changes in fishing stocks. This overlap of signals may obscure the perception of gradual declines in species abundance and diversity, constituting a second key factor behind the perceived absence of threats. This phenomenon may lead to delayed responses to biodiversity loss (Essl et al. 2015), particularly in contexts where changes in species abundance and diversity go unnoticed by local communities due to shifting baselines (i.e. situations in which reference conditions are gradually redefined over time) (Papworth et al. 2009; Daw 2010; Soga and Gaston 2018). This delayed perception of environmental change and threats may exacerbate existing issues, such as the impacts of climate change, overfishing, and food insecurity. Additionally, the absence of perceived threats may also be influenced by optimism bias (Weinstein 1987), where individuals tend to unrealistically imagine a low-risk future for themselves. In risk perception literature, it is common to observe a tendency to overestimate self-assessed abilities, particularly when individuals compare themselves to others perceived to be at greater risk (Joffe 2003).

Local sociocultural dynamics may further shape these perceptions, particularly in communities where fishing identity, attachment to place, and intergenerational continuity are central to social cohesion. In such contexts, acknowledging environmental degradation may conflict with narratives of resilience and stability, making the absence of perceived threat a socially embedded response rather than a simple lack of awareness (Farny and Dentoni 2025).

It is also important to consider that the difficulty in identifying threats may not stem solely from local perception but also from limitations in data collection. Although the qualitative approach adopted in the interviews was sufficient to capture fishers’ perceptions, it could be improved with more targeted questions capable of better distinguishing between fishers’ understanding of expected seasonal variations and a lack of perception of gradual ecological changes.

### Pressures on Livelihoods and Gendered Experiences

Regarding the socioeconomic variables associated with the perception of socio-environmental threats, we found significance for gender and income. Our study reinforces the findings in the literature that different perceptions of the environment can be understood in light of the socioeconomic conditions of small-scale fishers, their livelihoods, and the characteristics of their fishing activities (Silva and Lopes 2015; Martins et al. 2018; dos Santos et al. 2024).

Male gender was significantly associated with a greater perception of threats, specifically concerning overfishing and pollution. This result could be explained by the social construction of occupational differences and involvement in fishing activities (Gehrig et al., 2018). The different types of fishing gear and techniques used in small-scale fishing (e.g., gill nets, lines, manual gathering, or the use of tools) may form distinct identities and create social networks that shape subgroups within the fishing community. The social interactions within these occupational subgroups lead to a shared perception of the environment, as they use similar fishing methods (Gehrig et al., 2018). Although our data indicate higher male participation in the fishing activities in the studied areas, it is important to recognize the gender-based division of labor in these coastal regions.

Socially and historically, males have been associated with operating in the “outer sea” or offshore areas, where they conduct boat-based fishing. Females, in turn, predominantly work in the “inner sea,” that is, in areas closer to land and home, such as rivers, mangroves, and beaches (Alicia and Hellebrandt 2019). Female fishers play a crucial role in shellfish gathering, significantly contributing to family sustenance (Mourão et al. 2020). Beyond occupational differences, the social context also shapes gender roles in fishing activities, particularly concerning the spatial and temporal limitations imposed on them. Shellfish gathering becomes their primary activity, largely due to the accessibility of sandbanks, which do not require boats or expensive equipment. This practice, in addition to being carried out near residences, allows female fishers to balance work with childcare responsibilities (a double or even triple workload)., highlighting how social and structural factors influence the gendered division of labor in small-scale fishing (Kleiber et al. 2015; Alicia and Hellebrandt 2019). While males tend to perceive environmental impacts primarily in terms of declines in catch volume (i.e., fish quantity), females more often express concern about the rising cost of living and the implications of environmental degradation for household food security (Alicia and Hellebrandt 2019).

Our results indicate that small-scale fishers in the PAs of APABRM, APAT, and REAG have low levels of education, a high average age, and low income. Most respondents live at or below the poverty line, according to the World Bank’s criteria (US$6.85 per day) (World Bank 2025). This reality mirrors that of other coastal regions in Brazil (Silva and Lopes 2015; Barros 2021; dos Santos et al. 2024) and comparable small-scale fisheries contexts elsewhere (McClanahan and Abunge 2020), particularly where similar configurations of livelihood dependence, socioeconomic vulnerability, and governance constraints are present. Such a context poses significant challenges to the management of common-pool resources due to the socio-ecological complexity, as the maintenance of traditional fishing practices and the livelihoods of fishing communities are highly sensitive to changes in coastal and marine ecosystem quality and the availability of resources for subsistence.

Fishers living in conditions of greater socioeconomic vulnerability, with lower income and greater dependence on fishing for subsistence, tend to be more sensitive to changes in marine ecosystems, as these changes directly affect their ability to support their families (Cinner et al. 2014). In contexts where populations depend directly on natural resources and live near PAs, these communities may experience improvements in well-being and, to some extent, poverty alleviation (Andam et al. 2010), especially when involved in conservation initiatives that yield positive socioeconomic outcomes (Oldekop et al. 2016). This relationship reinforces the importance of incorporating human dimensions as central components in the planning and management of PAs, directing programs and policies specifically toward beneficiary communities while recognising local needs and social dynamics (Chechina et al. 2018)

One possible explanation for the absence of significance in the exclusive dependence on fishing as a source of income is the importance of income supplementation for households, particularly through government assistance. The results of our study indicate that fishers with lower family income tend to perceive environmental threats more acutely, while those with higher incomes do not show the same level of sensitivity. However, the majority of respondents consider fishing their primary source of income, in addition to receiving government assistance, which, while important, is not enough to lift them out of poverty, according to World Bank criteria (World Bank 2025). The condition of material insecurity, especially among individuals who rely directly on natural resources for subsistence, tends to contribute to a heightened perception of environmental risk (Htun et al. 2012; Lo 2014).

The relationship of these communities with nature extends beyond economic dimensions. These traditional populations in Northeastern Brazil rely on natural resources to ensure food sovereignty and decent livelihoods, while maintaining a strong commitment to sociobiodiversity. However, poverty disrupts this balance by constraining the freedoms necessary for people to lead the lives they value, including access to healthcare, education, and environmental protection (Barros 2021). Poverty and inequality scenarios highlight the importance of maintaining existing social policies, as well as the need to develop new strategies that combine social protection with the promotion of traditional small-scale fishing practices. In addition to the common social benefits of the Brazilian social security system (e.g., pensions, retirement, and unemployment benefits), the fishing sector also benefits from specific aids for this category of labor, such as payments during the closed season for certain species, such as lobster during its reproductive period (Barros 2021).

These perspectives connect to broader debates on poverty, demographic pressure, and resource depletion in small-scale fisheries. Hardin’s (1968) “tragedy of the commons” framed overexploitation as the outcome of individually rational behavior in open-access systems, often used to justify regulatory control or privatization as primary solutions. However, subsequent empirical work has demonstrated that collective action can sustain common-pool resources under specific institutional conditions (Ostrom et al. 1999). Building on this shift, small-scale fisheries frequently operate as poverty safety nets, indicating that effective governance must address structural vulnerability alongside resource management (Béné and Friend 2011).

Since the interviewed fishers have similar levels of education and age, these characteristics may have influenced the absence of significance in their perception of threats (dos Santos et al. 2024). These results highlight that, in this specific socio-territorial context, differences in threat perception are more strongly structured by livelihood conditions and gendered experiences than by generational or educational variation.

## Conclusion

This study shows that perceptions of fishing dynamics and socio-environmental change are shaped by income, and gendered experiences in coastal protected areas of northeastern Brazil. Overfishing was identified as the main perceived threat, even within protected areas, highlighting tensions between conservation objectives and local subsistence needs. Our results are aligned with empirical data on the global depletion of fish stocks. Technological advancements, such as modernization and greater access to equipment, despite increasing the efficiency of target species capture, bring long-term consequences, such as greater pressure on fishery resources. This perception, coupled with the persistence of predatory practices and a limited understanding of gradual changes in biodiversity, raises questions about the effectiveness of PAs in achieving biodiversity conservation objectives, the sustainable use of natural resources, and the protection of the livelihoods of traditional communities.

Lower income levels were associated with greater sensitivity to socio-environmental threats, while gendered divisions of labor influence distinct perceptions. These dynamics reveal how socio-ecological complexity in coastal protected areas is deeply intertwined with structural inequalities. By integrating ecological perceptions with socioeconomic conditions, this study underscores the need for governance approaches that address poverty, gender inequities, and livelihood security alongside biodiversity conservation. Inclusive and context-sensitive policies are essential to sustaining small-scale fisheries and reducing socio-environmental vulnerability.

## Supplementary information

Supplementary information

Supplementary information

Supplementary information

Supplementary information

Supplementary information

Supplementary information

## Data Availability

The datasets generated and/or analyzed during the current study are not publicly available due to confidentiality agreements with participants but are available from the corresponding author on reasonable request.

## References

[CR1] Agresti A (2018) An Introduction to Categorical Data Analysis, 3rd ed. Wiley

[CR2] Albuquerque UP, da Cunha LVFC, de Lucena RFP, Alves RRN (2014) Methods and Techniques in Ethnobiology and Ethnoecology. Springer Protocols Handbooks, New York

[CR3] Aldon MET, Fermin AC, Agbayani RF (2011) Socio-cultural context of fishers’ participation in coastal resources management in Anini-y, Antique in west central Philippines. Fish Res 107:112–121. 10.1016/j.fishres.2010.10.014.

[CR4] Alicia S, Hellebrandt L (2019) Mulheres na Atividade Pesqueira no Brasil. EDUENF, Campos dos Goytacazes, RJ

[CR5] Almeida NV (2006) Proposta de Zoneamento Ecológico Econômico para a Área de Proteção Ambiental (APA) Estadual de Tambaba-Paraíba. Universidade Federal da Paraíba

[CR7] Andam KS, Ferraro PJ, Sims KRE et al. (2010) Protected areas reduced poverty in Costa Rica and Thailand. Proc Natl Acad Sci USA 107:9996–10001. 10.1073/pnas.0914177107. 20498058 10.1073/pnas.0914177107 PMC2890456

[CR8] Baquiano MJ (2016) Understanding coastal resource management using a social representations approach. Ocean Coast Manag 133:18–27. 10.1016/j.ocecoaman.2016.09.008.

[CR9] Bardin L (2011) Análise de conteúdo. Traduzido por Luís Antero Reto, 1a. Edições 70, São Paulo

[CR10] Barros KRA (2021) Social Development and Sustainable Fisheries: Brazil. International Collective in Support of Fishworkers (ICSF), Tamil Nadu, India

[CR80] Béné C, Friend RM (2011) Poverty in small-scale fi sheries: old issue, new analysis. Prog Dev Stud 11:119–44.

[CR11] Bengtsson M (2016) How to plan and perform a qualitative study using content analysis. NursingPlus Open 2:8–14. 10.1016/j.npls.2016.01.001.

[CR12] Bennett NJ (2016) Using perceptions as evidence to improve conservation and environmental management. Conserv Biol 30:582–592. 10.1111/cobi.12681. 26801337 10.1111/cobi.12681

[CR13] Bennett NJ, Katz L, Yadao-Evans W et al. (2021) Advancing Social Equity in and Through Marine Conservation. Front Mar Sci 8:1–13. 10.3389/fmars.2021.711538.35685121

[CR14] Borges R, Eyzaguirre I, Barboza RSL, Glaser M (2020) Systematic Review of Spatial Planning and Marine Protected Areas: A Brazilian Perspective. Front Mar Sci 7:1–16. 10.3389/fmars.2020.00499.32802822

[CR15] BRASIL (2000) Lei Federal No 9.985, de 18 de julho de 2000. Regulamenta o art. 225, § 1o, incisos I, II, III e VII da Constituição Federal, institui o Sistema Nacional de Unidades de Conservação da Natureza e dá outras providências

[CR16] BRASIL (2009) Lei n° 11.959, de 29 de junho de 2009. Dispõe sobre a Política Nacional de Desenvolvimento Sustentável da Aquicultura e da Pesca, regula as atividades pesqueiras, revoga a Lei no 7.679, de 23 de novembro de 1988, e dispositivos do Decreto-Lei no 221, de 28 de fevereiro de 1967, e dá outras providências

[CR17] Burbano DV, Meredith TC (2020) Conservation Strategies Through the Lens of Small-Scale Fishers in the Galapagos Islands, Ecuador: Perceptions Underlying Local Resistance to Marine Planning. Soc Nat Resour 33:1194–1212. 10.1080/08941920.2020.1765058.

[CR18] Campbell LM, Gray NJ, Hazen EL, Shackeroff JM (2009) Beyond Baselines: Rethinking Priorities for Ocean Conservation. Ecol Soc 14:14. http://www.ecologyandsociety.org/vol14/iss1/art14/merits.

[CR19] Castello L, Viana JP, Watkins G et al. (2009) Lessons from Integrating Fishers of Arapaima in Small-Scale Fisheries Management at the Mamirauá Reserve, Amazon. Environ Manag 43:197–209. 10.1007/s00267-008-9220-5. 10.1007/s00267-008-9220-5 18946698

[CR20] Chambon M, Ziveri P, Alvarez Fernandez S et al. (2024) The gendered dimensions of small-scale fishing activities: A case study from coastal Kenya. Ocean Coast Manag 257: 107293. 10.1016/j.ocecoaman.2024.107293.

[CR21] Chechina M, Neveux Y, Parkins J, Hamann A (2018) Balancing conservation and livelihoods: A study of forest-dependent communities in the Philippines. Conserv Soc 16:420–430. 10.4103/cs.cs_16_182.

[CR22] Cinner JE, Daw T, Huchery C et al. (2014) Winners and losers in marine conservation: Fishers’ displacement and livelihood benefits from marine reserves. Soc Nat Resour 27:994–1005. 10.1080/08941920.2014.918229.

[CR23] Costello C, Ovando D, Hilborn R et al. (2012) Status and solutions for the world’s unassessed fisheries. Science (80-) 338:517–520. 10.1126/science.1223389. 10.1126/science.1223389 23019613

[CR24] Daw TM (2010) Shifting baselines and memory illusions: What should we worry about when inferring trends from resource user interviews?. Anim Conserv 13:534–535. 10.1111/j.1469-1795.2010.00418.x.

[CR25] Dean AJ, Wilson KA (2023) Relationships between hope, optimism, and conservation engagement. Conserv Biol 37:1–13. 10.1111/cobi.14020. 10.1111/cobi.14020 36285591

[CR6] de Alves MFP, Casaleiro PTA, Valentim ICSG et al. (2021) Viver do mar - caracterização socioeconómica das comunidades piscatórias de arte xávega em Portugal. RECIMA21 - Rev Científica Multidiscip - ISSN 2675-6218 2:e29633. 10.47820/recima21.v2i9.633.

[CR26] Essl F, Dullinger S, Rabitsch W et al. (2015) Delayed biodiversity change: No time to waste. Trends Ecol Evol 30:375–378. 10.1016/j.tree.2015.05.002. 26028440 10.1016/j.tree.2015.05.002

[CR27] Fadigas ABdeM, Garcia LG (2010) Uma análise do processo participativo para a conservação do ambiente na criação da Reserva Extrativista Acaú-Goiana. Soc Nat 22:561–576. 10.1590/s1982-45132010000300012.

[CR28] Farny S, Dentoni D (2025) Social identity and place-based dynamics in community resilience building for natural disasters: an integrative framework. Ecol Soc 30:12.

[CR29] Fernández-Llamazares Á, Díaz-Reviriego I, Luz AC et al. (2015) Rapid ecosystem change challenges the adaptive capacity of Local Environmental Knowledge. Glob Environ Chang 31:272–284. 10.1016/j.gloenvcha.2015.02.001. 10.1016/j.gloenvcha.2015.02.001 PMC4471143 26097291

[CR30] Freitas CT, Espírito-Santo HMV, Campos-Silva JV et al. (2020) Resource co-management as a step towards gender equity in fisheries. Ecol Econ 176: 106709. 10.1016/j.ecolecon.2020.106709.

[CR31] Gehrig S, Schlüter A, Jiddawi NS (2018) Overlapping identities: The role of village and occupational group for small-scale fishers’ perceptions on environment and governance. Mar Policy 96:100–110. 10.1016/j.marpol.2018.06.017.

[CR32] Goodman LA (1961) Snowball sampling. Ann Math Stat 32:148–170. 10.1214/aoms/1177705148.

[CR81] Greene W (2018) Econometric Analysis, 8th Edition. Pearson Education Limited, London

[CR33] Hardin G (1968) The tragedy of the commons. Science 162:1243–1248. 10.1126/science.162.3859.1243. 10.1126/science.162.3859.1243 17756331

[CR34] Heidari S, Babor TF, De Castro P et al. (2016) Sex and Gender Equity in Research: rationale for the SAGER guidelines and recommended use. Res Integr Peer Rev 1: 2. 10.1186/s41073-016-0007-6. 29451543 10.1186/s41073-016-0007-6 PMC5793986

[CR35] Hosmer DW, Lemeshow S, Sturdivant RX (2013) Applied Logistic Regression. Wiley

[CR36] Htun NZ, Mizoue N, Yoshida S (2012) Determinants of Local People’s Perceptions and Attitudes Toward a Protected Area and Its Management: A Case Study From Popa Mountain Park, Central Myanmar. Soc Nat Resour 25:743–758. 10.1080/08941920.2011.620597.

[CR37] Joffe H (2003) Risk: From perception to social representation. Br J Soc Psychol 42:55–73. 10.1348/014466603763276126. 12713756 10.1348/014466603763276126

[CR38] Juvan E, Dolnicar S (2016) Measuring environmentally sustainable tourist behaviour. Ann Tour Res 59:30–44. 10.1016/j.annals.2016.03.006.

[CR39] Kahn Jr PH (2002) Children’s affiliations with nature: Structure, development, and the problem of environmental generational amnesia. In: Kahn Jr P. H., Kellert S. R. (eds) Children and nature: Psychological, sociocultural, and evolutionary investigations. MIT Press., pp 93–116

[CR40] Karper MAM, Lopes PFM (2014) Punishment and compliance: Exploring scenarios to improve the legitimacy of small-scale fisheries management rules on the Brazilian coast. Mar Policy 44:457–464. 10.1016/j.marpol.2013.10.012.

[CR41] Kleiber D, Harris LM, Vincent ACJ (2015) Gender and small-scale fisheries: A case for counting women and beyond. Fish Fish 16:547–562. 10.1111/faf.12075.

[CR82] Long JS, Freese J (2014) Regression Models for Categorical Dependent Variables Using Stata, 3rd ed. Stata Press,Texas.

[CR42] Lo AY (2014) Negative income effect on perception of long-term environmental risk. Ecol Econ 107:51–58. 10.1016/j.ecolecon.2014.08.009.

[CR43] Marchal P, Andersen B, Caillart B et al. (2007) Impact of technological creep on fishing effort and fishing mortality, for a selection of European fleets. ICES J Mar Sci 64:192–209. 10.1093/icesjms/fsl014.

[CR44] Martins IM, Medeiros RP, Di Domenico M, Hanazaki N (2018) What fishers’ local ecological knowledge can reveal about the changes in exploited fish catches. Fish Res 198:109–116. 10.1016/j.fishres.2017.10.008.

[CR45] Mcalvay AC, Armstrong CG, Baker J et al. (2021) Ethnobiology Phase VI: Decolonizing Institutions, Projects, and Scholarship. J Ethnobiol 41:170–191. 10.2993/0278-0771-41.2.170.

[CR46] McClanahan T, Abunge C (2020) Perceptions of governance effectiveness and fisheries restriction options in a climate refugia. Biol Conserv 246: 108585. 10.1016/j.biocon.2020.108585.

[CR47] Medeiros IS, Santos SS, Rebelo VA et al. (2023) Effectiveness of Federal Protected Areas in the Preservation of Mangrove Forests on the Coast of the State of Paraíba, Brazil. Acad Bras Cienc 95:1–15. 10.1590/0001-3765202320211079. 10.1590/0001-3765202320211079 37222361

[CR48] Medeiros MC, Barboza RRD, Martel G, Mourão JdaS (2018) Combining local fishers’ and scientific ecological knowledge: Implications for comanagement. Ocean Coast Manag 158:1–10. 10.1016/j.ocecoaman.2018.03.014.

[CR49] Medeiros MC, Silva Pinto A, de Morais Rodrigues E, et al (2024) Changes in artisanal fishing as a driver of the shifting baseline syndrome: from the perspective of local ecological knowledge. Mar Policy 165. 10.1016/j.marpol.2024.106204

[CR50] Moscovici S (1976) La psychanalyse, son image et son public. Presses Universitaires de France, Paris, France

[CR51] da Mourão JS, Nordi N (2018) Etnoictiologia de pescadores artesanais do estuário do rio Mamanguape, Paraíba, Brasil. Bol do Inst Pesca 29:9–17.

[CR52] da Silva Mourãoda J, Baracho RL, Martel G et al. (2020) Local ecological knowledge of shellfish collectors in an extractivist reserve, Northeast Brazil: implications for co-management. Hydrobiologia 847:1977–1997. 10.1007/s10750-020-04226-w.

[CR53] Mudge L (2018) Use of community perceptions to evaluate and adapt coastal resource management practices in the Philippines. Ocean Coast Manag 163:304–322. 10.1016/j.ocecoaman.2018.07.008.

[CR54] Oldekop JA, Holmes G, Harris WE, Evans KL (2016) A global assessment of the social and conservation outcomes of protected areas. Conserv Biol 30:133–141. 10.1111/cobi.12568. 26096222 10.1111/cobi.12568

[CR55] Ostrom E, Burger J, Field CB et al. (1999) Revisiting the commons: Local lessons, global challenges. Science (80-) 284:278–282. 10.1126/science.284.5412.278. 10.1126/science.284.5412.278 10195886

[CR56] Panagopoulou A, Meletis ZA, Margaritoulis D, Spotila JR (2017) Caught in the same net? Small-scale fishermen’s perceptions of fisheries interactions with sea turtles and other protected species. Front Mar Sci 4:1–15. 10.3389/fmars.2017.00180.

[CR57] Papworth SK, Rist J, Coad L, Milner-Gulland EJ (2009) Evidence for shifting baseline syndrome in conservation. Conserv Lett 2:93–100. 10.1111/j.1755-263X.2009.00049.x.

[CR58] Pauly D (1995) Anecdotes and the shifting baseline syndrome of fisheries. Trends Ecol Evol 10:430. 10.1016/S0169-5347(00)89171-5. 21237093 10.1016/s0169-5347(00)89171-5

[CR59] Perazzo ARF, de Meneses LFI, Cavalcante MB (2013) Etnogeodiversidade Em Comunidade Tradicional Da Barra Do Rio Mamanguape, Município De Rio Tinto, Paraíba. Bras Rev Ouricuri 3:01–18.

[CR60] Pita C, Villasante S, Pascual-Fernández JJ (2019) Managing small-scale fisheries under data poor scenarios: lessons from around the world. Mar Policy 101:154–157. 10.1016/j.marpol.2019.02.008.

[CR61] R Core Team (2023) R: A language and environment for statistical computing. In: R Found. Stat. Comput. https://www.r-project.org/.

[CR62] Rodrigues GG, Souza AEVN, Lima MEA et al. (2017) Território, paisagens e identidades culturais em uma Reserva Extrativista marinha do nordeste brasileiro. Rev Mov Sociais Dinâmicas Espac 06:235–242.

[CR63] Sandoval Gallardo S, Fossile T, Herbst DF et al. (2021) 150 years of anthropogenic impact on coastal and ocean ecosystems in Brazil revealed by historical newspapers. Ocean Coast Manag 209: 105662. 10.1016/j.ocecoaman.2021.105662.

[CR64] dos Santos AO, Oliveira CDL, de Oliveira Junior JGC, da Silva Batista V (2024) Factors influencing risk perception of inshore and offshore artisanal fishers in a marine protected area in Brazil. Fish Manag Ecol 31:1–11. 10.1111/fme.12666.

[CR65] Seixas CS, Kalikoski DC (2009) Gestão participativa da pesca no Brasil: levantamento das iniciativas e documentação dos processos. Desenvolv e Meio Ambient 20:119–139. 10.5380/dma.v20i0.12729.

[CR66] Seixas CS, Kalikoski DC, Almudi T et al. (2011) Gestão compartilhada do uso de recursos pesqueiros no Brasil: elementos para um programa nacional. Ambient Soc 14:23–44. 10.1590/S1414-753X2011000100003.

[CR67] Silva MRO, Lopes PFM (2015) Each fisherman is different: Taking the environmental perception of small-scale fishermen into account to manage marine protected areas. Mar Policy 51:347–355. 10.1016/j.marpol.2014.09.019.

[CR68] Silva MRO, Pennino MG, Lopes PFM (2021) Predicting potential compliance of small-scale fishers in Brazil: The need to increase trust to achieve fisheries management goals. J Environ Manag 288: 112372. 10.1016/j.jenvman.2021.112372. 10.1016/j.jenvman.2021.112372 33756387

[CR69] da Silveira MF, Ferreira BP (2024) Temporal changes in a small-scale artisanal reef fishery in Brazil: Coastal development and its impacts. Mar Policy 165: 106186. 10.1016/j.marpol.2024.106186.

[CR70] Soga M, Gaston KJ (2018) Shifting baseline syndrome: causes, consequences, and implications. Front Ecol Environ 16:222–230. 10.1002/fee.1794.

[CR71] Sowman M, Sunde J (2018) Social impacts of marine protected areas in South Africa on coastal fishing communities. Ocean Coast Manag 157:168–179. 10.1016/j.ocecoaman.2018.02.013.

[CR72] Tallman RF, Roux MJ, Martin ZA (2019) Governance and assessment of small-scale data-limited Arctic Charr fisheries using productivity-susceptibility analysis coupled with life history invariant models. Mar Policy 101:187–197. 10.1016/j.marpol.2017.11.032.

[CR73] Teh L, Sumaila U (2007) Malthusian overfishing in Pulau Banggi?. Mar Policy 31:451–457. 10.1016/j.marpol.2007.01.001.

[CR74] Torres-Irineo E, Gaertner D, Chassot E, Dreyfus-león M (2014) Changes in fishing power and fishing strategies driven by new technologies: The case of tropical tuna purse seiners in the eastern Atlantic Ocean. Fish Res 155:10–19. 10.1016/j.fishres.2014.02.017.

[CR75] Van Epps H, Astudillo O, Del Pozo Martín Y, Marsh J (2022) The Sex and Gender Equity in Research (SAGER) Guidelines: Implementation and Checklist Development, E86910 edn 10.12771/emj.2024.e11 PMC12093626 40703401

[CR76] Viegas V, Azeiteiro UM, Alves F (2016) Fostering Resilience Among Artisanal Fishers in Peniche (Portugal): An Exploratory Study. In: Leal Filho W., Musa H., Cavan G. et al. (eds) Climate Change Adaptation, Resilience and Hazards. Climate Change Management. Springer, Cham, pp 305–327

[CR77] Vieira S, Jiménez V, Ferreira-Airaud B et al. (2024) Perceived social benefits and drawbacks of sea turtle conservation efforts in a globally important sea turtle rookery. Biodivers Conserv 33:1185–1205. 10.1007/s10531-024-02793-1.

[CR78] Weinstein ND (1987) Unrealistic optimism about susceptibility to health problems: Conclusions from a community-wide sample. J Behav Med 10:481–500. 10.1007/BF00846146. 3430590 10.1007/BF00846146

[CR79] World Bank (2025) Poverty: Development news, research, data. In: World Bank Gr. https://www.worldbank.org/en/topic/poverty. Accessed 3 Jan 2025

