# Supplementary material for: Fishers’ Perceptions of Fishing Dynamics and Socio-environmental Threats in Coastal Protected Areas of Northeastern Brazil
Source: Environ Manage. 2026 Apr 16;76(5):161. doi: 10.1007/s00267-026-02465-6 (PMC13086653; doi:10.1007/s00267-026-02465-6)
Supplement: Supplementary file 3 — Supplementary information [file 267_2026_2465_MOESM3_ESM.docx]

**Supplementary Material Information – S3**

**Article Title:** Fishers' perceptions of fishing dynamics and socio-environmental threats in coastal protected areas of northeastern Brazil

**Journal:** Environmental Management

**Authors and Affiliations:**

**Yedda Christina Bezerra Barbosa de Oliveira**
Researcher, Programa de Pós-Graduação em Etnobiologia e Conservação da Natureza, Universidade Federal
Researcher, Centre for Functional Ecology (CFE), Universidade de Coimbra,
E-mail: yedda.oliveira@gmail.com

**Priscila Fabiana Macedo Lopes**
Associate Professor, Departamento de Ecologia, Universidade Federal do Rio Grande do Norte,
Researcher, Research Institute of the University of Bucharest,
Researcher, Institute of Biological Research Cluj, National Institute of Research and Development for Biological Sciences,

**Tiago Almeida de Oliveira**
Associate Professor, Departamento de Estatística, Universidade Estadual da Paraíba,

**Diogo Guedes Vidal**
Researcher, Centre for Functional Ecology (CFE), Universidade de Coimbra,
Assistant Professor, Department of Social Sciences and Management, Universidade Aberta,

**Maria de Fátima Pereira Alves**
Associate Professor, Department of Social Sciences and Management, Universidade Aberta,
Researcher, Centre for Functional Ecology (CFE), Universidade de Coimbra,

**Maria do Rosário Tomás Rosa**
Assistant Professor, Department of Social Sciences and Management, Universidade Aberta,
Researcher, Centre for Functional Ecology (CFE), Universidade de Coimbra,
Calçada Martim de Freitas, 3000-456 Coimbra, Portugal.

**José da Silva Mourão**
Associate Professor, Departamento de Biologia, Universidade Estadual da Paraíba,
Associate Professor, Programa de Pós-Graduação em Etnobiologia e Conservação da Natureza, Universidade


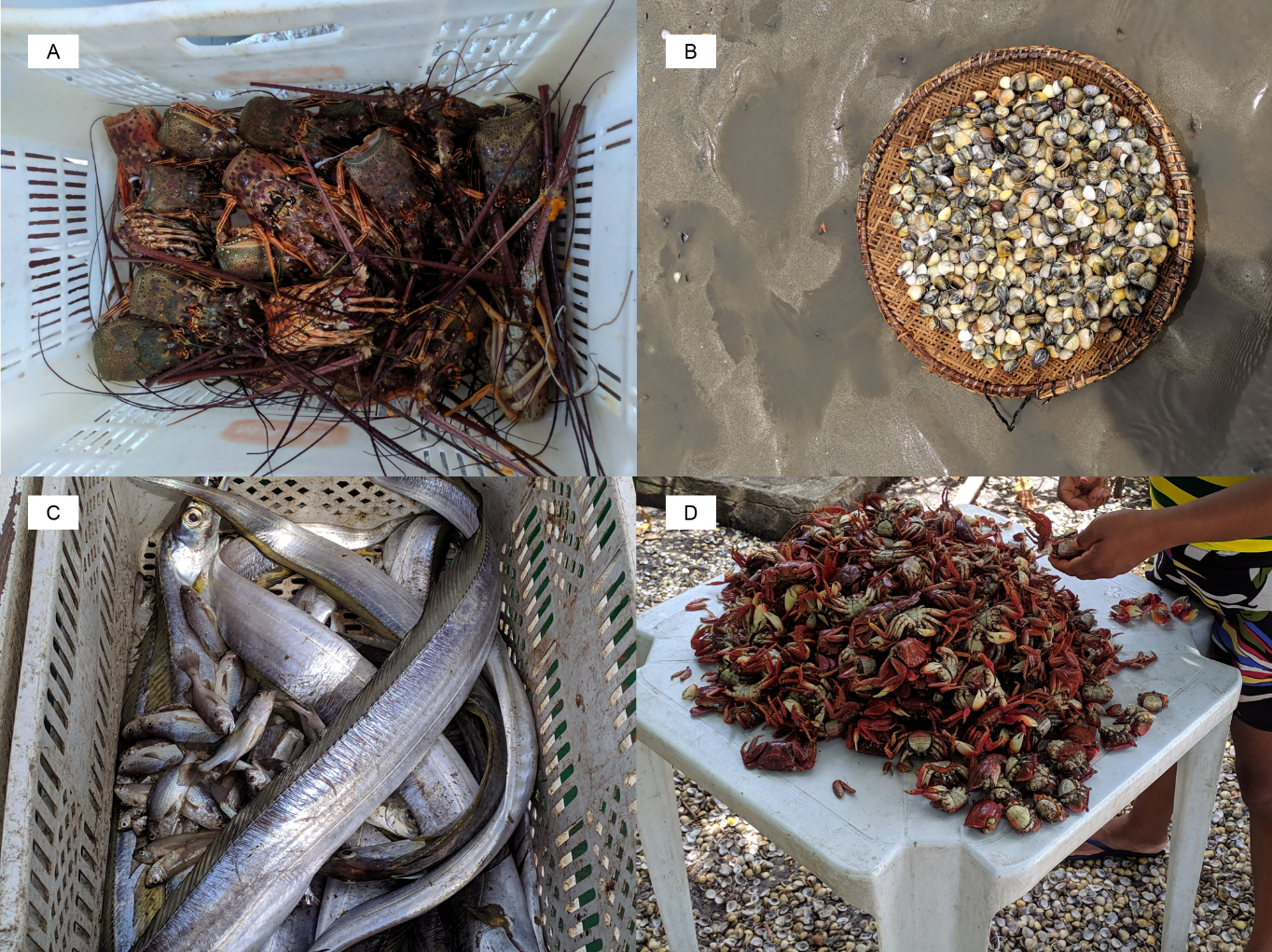
**Figure S3.** Examples of fishery resources extracted from the studied coastal protected areas in Paraíba and Pernambuco. (A) Panulirus argus. (B) Anomalocardia flexuosa. (C) Xiphias gladius and Macrodon ancylodon. (D) Goniopsis cruentata.
