# Supplementary material for: Fishers’ Perceptions of Fishing Dynamics and Socio-environmental Threats in Coastal Protected Areas of Northeastern Brazil
Source: Environ Manage. 2026 Apr 16;76(5):161. doi: 10.1007/s00267-026-02465-6 (PMC13086653; doi:10.1007/s00267-026-02465-6)
Supplement: Supplementary file 4 — Supplementary information [file 267_2026_2465_MOESM4_ESM.docx]

**Supplementary Material Information – S4**

**Article Title:** Fishers' perceptions of fishing dynamics and socio-environmental threats in coastal protected areas of northeastern Brazil

**Journal:** Environmental Management

**Authors and Affiliations:**

**Yedda Christina Bezerra Barbosa de Oliveira**
Researcher, Programa de Pós-Graduação em Etnobiologia e Conservação da Natureza, Universidade Federal
Researcher, Centre for Functional Ecology (CFE), Universidade de Coimbra,
E-mail: yedda.oliveira@gmail.com

**Priscila Fabiana Macedo Lopes**
Associate Professor, Departamento de Ecologia, Universidade Federal do Rio Grande do Norte,
Researcher, Research Institute of the University of Bucharest,
Researcher, Institute of Biological Research Cluj, National Institute of Research and Development for Biological Sciences,

**Tiago Almeida de Oliveira**
Associate Professor, Departamento de Estatística, Universidade Estadual da Paraíba,

**Diogo Guedes Vidal**
Researcher, Centre for Functional Ecology (CFE), Universidade de Coimbra,
Assistant Professor, Department of Social Sciences and Management, Universidade Aberta,

**Maria de Fátima Pereira Alves**
Associate Professor, Department of Social Sciences and Management, Universidade Aberta,
Researcher, Centre for Functional Ecology (CFE), Universidade de Coimbra,

**Maria do Rosário Tomás Rosa**
Assistant Professor, Department of Social Sciences and Management, Universidade Aberta,
Researcher, Centre for Functional Ecology (CFE), Universidade de Coimbra,
Calçada Martim de Freitas, 3000-456 Coimbra, Portugal.

**José da Silva Mourão**
Associate Professor, Departamento de Biologia, Universidade Estadual da Paraíba,
Associate Professor, Programa de Pós-Graduação em Etnobiologia e Conservação da Natureza, Universidade

**Table S4.** Information and questions from the semi-structured interview with fishers from the three protected areas: Tambaba Environmental Protection Area (APA), Barra do Rio Mamanguape APA, and Acaú-Goiana Extractive Reserve.

PART 1 – INTERVIEWEE IDENTIFICATION

PA: ( ) Tambaba APA ( ) Barra do Rio Mamanguape APA ( ) Acaú-Goiana RESEX
ID:
Sex: ( ) M ( ) F Age:

PART 2 – PERCEPTIONS ABOUT FISHING

1. At what age did you start fishing?
2. Fishing location: ( ) River/Estuary ( ) Inshore ( ) Offshore ( )
3. How many days a week do you fish?
4. Have you noticed any changes in fishing over time? ( ) Yes ( ) No, What changes?
5. What type of vessel do you use?
6. What types of fishing gear do you use?
7. Are these the same gears you used when you started fishing? ( ) Yes ( ) No, Why?

PART 3 – SOCIOECONOMICS

1. What is your level of education?
   ( ) Can write their name ( ) Incomplete High School (1st to 2nd year)
   ( ) Incomplete Elementary School I (1st to 3rd) ( ) Completed High School (3rd year)
   ( ) Completed Elementary School I (4th) ( ) Technical Course
   ( ) Incomplete Elementary School II (5th to 7th) ( ) Higher Education
   ( ) Completed Elementary School II (8th)
   ( ) Cannot read ( ) No answer
2. Is your house:
   ( ) Owned ( ) Rented ( ) Given
3. How many people live in your house? ________________ What is the family income source? (Fishing, Tourism, Retirement) ____________________ How many household members contribute to this income? ___________
4. What is your family income?
   ( ) up to one thousand reais (<= USD 250) ( ) More than one thousand up to two thousand reais (> USD 250 to USD 500)
   ( ) More than two thousand up to three thousand reais (> USD 500 to USD 750) ( ) More than three thousand reais (> USD 750)
   /Purchasing Power Parities (PPP) x 0.25 US Dollar (USD)
5. Is fishing your main source of income? And how long have you been a fisher or shellfisher?
6. Is there any other income activity dependent on the sea/coastal zone? ( ) Yes ( ) No / If yes, what?
