# Supplementary material for: Fishers’ Perceptions of Fishing Dynamics and Socio-environmental Threats in Coastal Protected Areas of Northeastern Brazil
Source: Environ Manage. 2026 Apr 16;76(5):161. doi: 10.1007/s00267-026-02465-6 (PMC13086653; doi:10.1007/s00267-026-02465-6)
Supplement: Supplementary file 5 — Supplementary information [file 267_2026_2465_MOESM5_ESM.docx]

**Supplementary Material Information – S5**

**Article Title:** Fishers' perceptions of fishing dynamics and socio-environmental threats in coastal protected areas of northeastern Brazil

**Journal:** Environmental Management

**Authors and Affiliations:**

**Yedda Christina Bezerra Barbosa de Oliveira**
Researcher, Programa de Pós-Graduação em Etnobiologia e Conservação da Natureza, Universidade Federal
Researcher, Centre for Functional Ecology (CFE), Universidade de Coimbra,
E-mail: yedda.oliveira@gmail.com

**Priscila Fabiana Macedo Lopes**
Associate Professor, Departamento de Ecologia, Universidade Federal do Rio Grande do Norte,
Researcher, Research Institute of the University of Bucharest,
Researcher, Institute of Biological Research Cluj, National Institute of Research and Development for Biological Sciences,

**Tiago Almeida de Oliveira**
Associate Professor, Departamento de Estatística, Universidade Estadual da Paraíba,

**Diogo Guedes Vidal**
Researcher, Centre for Functional Ecology (CFE), Universidade de Coimbra,
Assistant Professor, Department of Social Sciences and Management, Universidade Aberta,

**Maria de Fátima Pereira Alves**
Associate Professor, Department of Social Sciences and Management, Universidade Aberta,
Researcher, Centre for Functional Ecology (CFE), Universidade de Coimbra,

**Maria do Rosário Tomás Rosa**
Assistant Professor, Department of Social Sciences and Management, Universidade Aberta,
Researcher, Centre for Functional Ecology (CFE), Universidade de Coimbra,
Calçada Martim de Freitas, 3000-456 Coimbra, Portugal.

**José da Silva Mourão**
Associate Professor, Departamento de Biologia, Universidade Estadual da Paraíba,
Associate Professor, Programa de Pós-Graduação em Etnobiologia e Conservação da Natureza, Universidade

**Table S5.** **Coding criteria for the content analysis of perceived changes in small-scale fisheries dynamics.**

| **Category** | **Operational definition** | **Inclusion criteria** | **Exclusion criteria** |
| --- | --- | --- | --- |
| **Decline in productivity** | Explicit comparison between past and present indicating reduction in catch volume, species abundance, size of individuals, or success rate per fishing trip. | Mentions of catching fewer kg than before; needing more effort for less return; disappearance or scarcity of species; reduced size of fish/shellfish; higher probability of returning to landing port empty. | Statements attributing change exclusively to seasonality without long-term comparison. |
| **Increase in productivity** | Explicit perception that catches volume or abundance has increased compared to the past. | Mentions of “there are more than/now”, “improved”, greater availability than before. | Isolated good fishing days without temporal comparison. |
| **Advancement in gear and technology** | Introduction, substitution, or diffusion of fishing gear, motorization, dredges, compressors, larger boats, or other technological changes altering capture method or effort. | Mentions of transition from hand gathering to gear; from sail to motor; new nets; increased efficiency due to equipment. | References only to increased number of fishers without mention of technological change. |
| **Predatory fishing practices** | Explicit identification of destructive capture methods that remove juveniles, damage habitats, or overexploit stocks. | Mentions of small mesh (e.g., 8 mm), dragging gear destroying substrate, harvesting undersized individuals. | General decline in fish without attribution to harmful practice. |
| **Increase in number of fishers** | Perceived growth in fishing pressure due to more boats, fishers, or community participation in fishing. | Mentions of “more boats”, “more fishers”, expansion of activity across households. | Technological intensification without explicit reference to number of actors. |
| **Changes in the landscape** | Perceived physical transformation of river, estuary, coastline, seabed or mangrove structure affecting fishing grounds. | References to silting, drying rivers, sea retreat/advance, sandbanks, erosion, habitat loss. | Biotic aspects (e.g., changes in species abundance, composition, or behavior) unless explicitly framed as physical alteration of landscape structure. |
| **Increased enforcement** | Perception of intensified state regulation or monitoring affecting fishing practices. | Mentions of IBAMA, Navy presence, bureaucracy, protected area influence, increased inspection. | General environmental concerns without reference to regulatory issues, informal social control. |
| **Devaluation of small-scale fishers’ labor** | Perceived decline in occupational prestige, intergenerational continuity, or viability of fishing as livelihood. | Statements about not wanting children to fish; youth migration to other sectors; profession “not worth it”. | Reduced income due solely to productivity decline without occupational framing. |
| **Increased value of catch** | Perceived increase in market price, commercialization opportunities, or better selling conditions independent of production volume. | Mentions of higher price per kg; selling at market value; better commercialization channels. | Higher income due solely to increased productivity. |
| **Invasive species** | Perceived appearance or increase of non-native species affecting ecosystem dynamics. | Explicit mention of lionfish or other non-native species. | Native species abundance change. |
| **Recovery of threatened species** | Perceived increase in protected or previously rare species. | Mentions of sea turtle increase or other endangered especies. | General biodiversity discussion without explicit increase. |
| **Seasonal variations** | Explicit denial of long-term change; variability attributed to natural cycles (e.g., winter/summer). | Statements such as “everything is the same”, “it's seasonal”, “it is always been like this”. | Statements containing both seasonal reference and long-term decline. |
