# Supplementary material for: Fishers’ Perceptions of Fishing Dynamics and Socio-environmental Threats in Coastal Protected Areas of Northeastern Brazil
Source: Environ Manage. 2026 Apr 16;76(5):161. doi: 10.1007/s00267-026-02465-6 (PMC13086653; doi:10.1007/s00267-026-02465-6)
Supplement: Supplementary file 6 — Supplementary information [file 267_2026_2465_MOESM6_ESM.docx]

**Supplementary Material Information – S6**

**Article Title:** Fishers' perceptions of fishing dynamics and socio-environmental threats in coastal protected areas of northeastern Brazil

**Journal:** Environmental Management

**Authors and Affiliations:**

**Yedda Christina Bezerra Barbosa de Oliveira**
Researcher, Programa de Pós-Graduação em Etnobiologia e Conservação da Natureza, Universidade Federal
Researcher, Centre for Functional Ecology (CFE), Universidade de Coimbra,
E-mail: yedda.oliveira@gmail.com

**Priscila Fabiana Macedo Lopes**
Associate Professor, Departamento de Ecologia, Universidade Federal do Rio Grande do Norte,
Researcher, Research Institute of the University of Bucharest,
Researcher, Institute of Biological Research Cluj, National Institute of Research and Development for Biological Sciences,

**Tiago Almeida de Oliveira**
Associate Professor, Departamento de Estatística, Universidade Estadual da Paraíba,

**Diogo Guedes Vidal**
Researcher, Centre for Functional Ecology (CFE), Universidade de Coimbra,
Assistant Professor, Department of Social Sciences and Management, Universidade Aberta,

**Maria de Fátima Pereira Alves**
Associate Professor, Department of Social Sciences and Management, Universidade Aberta,
Researcher, Centre for Functional Ecology (CFE), Universidade de Coimbra,

**Maria do Rosário Tomás Rosa**
Assistant Professor, Department of Social Sciences and Management, Universidade Aberta,
Researcher, Centre for Functional Ecology (CFE), Universidade de Coimbra,
Calçada Martim de Freitas, 3000-456 Coimbra, Portugal.

**José da Silva Mourão**
Associate Professor, Departamento de Biologia, Universidade Estadual da Paraíba,
Associate Professor, Programa de Pós-Graduação em Etnobiologia e Conservação da Natureza, Universidade

**Table S6.** **Coding criteria for the content analysis of perceived socio-environmental threats.**

| **Category** | **Operational definition** | **Inclusion criteria** | **Exclusion criteria** |
| --- | --- | --- | --- |
| **Overfishing** | Underlying perception that resource depletion results from excessive extraction pressure, intensified effort, technological efficiency, or harmful fishing methods. | Narratives linking decline to “too much people fishing”, small mesh nets, dredging, habitat destruction by gear, excessive boats. | Decline attributed solely to climate, pollution, or landscape change without fishing pressure reference. |
| **Pollution** | Perceived contamination of water or ecosystem due to industrial discharge, shrimp farms, sewage, oil, waste or chemical runoff affecting fish stocks. | Mentions of industrial chemical waste, trash, dirty water, aquaculture effluents. | Natural sedimentation not linked to contamination. |
| **Climate change, land use and occupation** | Perceived environmental changes attributed to climatic variability (e.g., shifts in sea level, temperature, or rainfall patterns) and/or to land-use and occupation processes, including urbanization, deforestation, and other territorial transformations. | Statements describing environmental changes attributed to climatic processes (e.g., increased heat, altered rainfall patterns, changes in river or ocean dynamics, salinity shifts) and/or environmental degradation associated with land-use and occupation processes (e.g., tourism growth, population increase, housing expansion, deforestation, or other territorial transformations affecting fishing grounds). | Statements referring solely to increased fishing effort or extraction pressure, without explicit reference to climatic processes or broader land-use and occupation dynamics. |
| **Food insecurity** | Perception that declining fishery productivity compromises subsistence and household food access. | Statements about not catching enough to eat; inability to sustain family; subsistence decline compared to past. | Income complaints not linked to food provision. |
| **Endangered biodiversity** | Perceived disappearance, rarity or local extinction of marine species beyond commercially targeted decline. | Mentions of coral decline, seahorses, turtles, manatees extinction framing. | Generic decline in fish abundance without biodiversity framing. |
| **No threat identified** | Explicit denial of environmental or fishing-related threats. | Statements indicating no perceived problem. | Ambiguous answers. |
